# Avian influenza A(H7N9) virus and mixed live poultry–animal markets in Guangdong province: a perfect storm in the making?

**DOI:** 10.1038/emi.2015.63

**Published:** 2015-10-14

**Authors:** Pei Zhou, Jun Ma, Alexander Lai, Gregory C Gray, Shuo Su, Shoujun Li

**Affiliations:** 1 College of Veterinary Medicine, South China Agricultural University, Guangzhou 510642, Guangdong Province, China; 2 Key Laboratory of Zoonosis Prevention and Control of Guangdong, Guangzhou 510642, Guangdong Province, China; 3 College of Arts and Sciences, Kentucky State University, Frankfort, KY 40601, USA; 4 Division of Infectious Diseases, Global Health Institute, Nicholas School of the Environment, Duke University, Durham, NC 27710 USA

## 

**Dear Editor**,

A new reassortant viral subtype of avian influenza virus named H7N9 was first identified in mainland China on March 27, 2013.^1^ Human cases of influenza A (H7N9) infections have continued to occur since then, with a larger ‘third wave' of cases occurring this last winter (2014–2015).^2,3^ As of May 9, 2015, 618 human A (H7N9) infections have been documented in China. Notably, most cases have occurred in Guangdong province (176 cases) at a seemingly accelerated rate. Thus far in 2015, 66 cases have been documented (Figure 1A). Whether this abrupt increase is a harbinger of a larger wave next winter remains to be seen; however, by comparing the previous two waves, we have found evidence for increased transmission during 2014–2015.^4^ Fortunately, although clusters or cases among family members have been reported, thus far there has been no evidence of sustained human-to-human transmission. Most of the cases have been associated with exposure to live poultry markets, and at these markets, A (H7N9) viruses have been almost exclusively detected in poultry.^2,5,6^

Similar to A (H5N1), genetic analysis of isolates from live poultry markets and from human cases of A (H7N9) has shown multiple regionalized lineages.^7^ This virus is now considered to be entrenched in China's poultry,^8^ increasing the risk of further adaptation and/or reassortment with seasonal influenza virus to create a pandemic virus. Furthermore, infection by A (H7N9) has not resulted in overt signs of disease in poultry, hampering surveillance, and intervention efforts. While the temporary closing of live animal markets has interrupted the reports of human cases,^9^ for cultural and economic reasons, permanently closing these markets is extremely difficult. Notably, unlike the situation with A (H5N1), which has spread to more than 16 countries through trade and by migratory birds, A (H7N9) has not been detected outside of China,^10^ which may be a result of the unique viral ecology in China.

Guangdong province is the most populous region in China, with an estimated population of 107.24 million persons in 2014. This province is home to the largest, and likely most species-diverse poultry industry in China, satisfying a huge demand. Additionally, outside of Guangdong province, large-scale broiler production and egg production facilities bring their products into Guangdong province. This large-scale movement of poultry and poultry products represents a huge risk of the continued spread of H7N9 virus among poultry stocks, as well as H7N9 infections among people. The high sequence similarity between the first H7N9 viruses identified in Guangdong province and previous detected strains from the Yangtze River Delta region suggests that the Guangdong H7N9 viruses were most likely unknowingly ‘imported' into Guangdong province from the Yangtze River Delta region. There are also many small backyard farms where chickens, pigs, ducks, geese, and passerine birds freely intermingle and roam within the farm boundaries (Figure 1C). Biosecurity in these small farms is largely non-existent. Additionally, Guangdong's live animal markets are unique (Figure 1D) in that, in support of the wide array of Chinese Cantonese cuisine, typical markets have live poultry (chickens, quail, ducks, and geese) in cages adjacent to numerous other small mammals (rabbits, ferrets, and piglets). Feral dogs and cats freely move between these cages foraging for food.^11,12,13^ In addition, Guangdong province is located in the flyway for migratory waterfowl, where the rice paddies provide places for migratory birds to stop, rest, and feed. In addition, Guangdong province is the year-round home to numerous species of wild birds. Hence, wild birds have frequent opportunities for direct contact with Guangdong livestock (especially on small farms). Correspondingly, southern China has earned the title of ‘epicenter for pandemic influenza,^14^ because of this unique viral ecosystem.

A careful study of the most recent human A (H7N9) cases in Guangdong province (source: http://www.gdwst.gov.cn/phact/search.php?keywords=H7N9&area=title) revealed that the number of locations (cities or small villages) with laboratory-confirmed human cases has increased and become more dispersed (Supplementary Table S1 and Figure 1B). This dispersion of cases is a cause for concern, as resources for diagnosis, medical care, and intervention in these rural areas are not as good as those in the larger metropolitan cities such as Guangzhou and Shenzhen. Making matters worse, the poultry trade in these rural areas is smaller, more dispersed, and difficult to track.

In an effort to develop a ‘proportional response' to the H7N9 epidemic,^15^ the Chinese government has intervened with numerous measures, such as temporarily closing live markets in large metropolitan cities (e.g., Guangzhou and Shenzhen) and encouraging the consumption of refrigerated processed poultry products to reduce human–poultry contact; however, the Chinese government still faces the dilemma of imposing more stringent regulations on live animal trade without encouraging underground trading. Nonetheless, we argue that more effort should be focused on (i) enforcing stringent segregation of species in these live markets; (ii) conducting more frequent and effective disinfection in these markets; (iii) establishing a more vigorous surveillance program for novel virus infections among the animals in these live markets; (iv) eliminating all free-ranging or stray dogs and cats from these live animal markets; and (v) imposing regular ‘resting days' in these markets. Given that these laboratory-confirmed human cases are likely just the tip of the iceberg, we strongly recommend that the Chinese authorities establish routine and broad serological surveillance among the poultry in these markets to assess the extent of A (H7N9) infection in the live markets and in farms supplying the poultry. In addition, surveillance for influenza-like-illness among the animal-exposed workers, particularly veterinarians, and free medical evaluations with viral etiology and serologic workups should be conducted. We have previously found elevated anti-H7 antibodies in such veterinarians.^16^ Live animal market workers and the veterinarians who care for these animals may be the first to be infected with novel influenza virus infections. While at this time an A (H7N9) pandemic does not seem imminent, the conditions described above certainly favor ‘a perfect storm' for the generation of novel influenza viruses. The employment of an interdisciplinary ‘One Health' approach in aggressively responding to these threats is imperative.

## Figures and Tables

**Figure 1:**
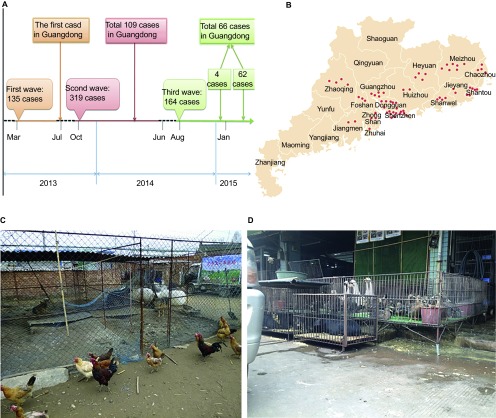
(**A**) Timeline of the human cases of A (H7N9) infection. (**B**) Distribution of the human cases of A (H7N9) infection in Guangdong province. (**C**) A backyard system in southern China. (**D**) A typical live, mixed, poultry–animal markets in Guangdong province.
