# Supplementary material for: Avian influenza A(H7N9) virus and mixed live poultry–animal markets in Guangdong province: a perfect storm in the making?
Source: Emerg Microbes Infect. 2015 Oct 14;4(10):e63–. doi: 10.1038/emi.2015.63 (PMC4631930; doi:10.1038/emi.2015.63)
Supplement: Supplementary Table S1 [file emi201563x1.docx]

**Supplementary Table S1** The characteristic of thelaboratory-confirmed human H7N9 casesin Guangdong Province.

| Number | Province / Region /Municipality | City / District /Area | Age (years) | Sex | Date of report |
| --- | --- | --- | --- | --- | --- |
|  |  |  |  |  |  |
| case1 | Guangdong | Shenzhen | 6 | F | 5/1/2015 |
| case2 | Guangdong | Dongguan | 36 | M | 6/1/2015 |
| case3 | Guangdong | Zhaoqing | 56 | M | 7/1/2015 |
| case4 | Guangdong | Shenzhen | 42 | M | 12/1/2015 |
| case5 | Guangdong | Dongguan | 52 | F | 12/1/2015 |
| case6 | Guangdong | Shenzhen | 57 | F | 13/1/2015 |
| case7 | Guangdong | Shenzhen | 41 | M | 13/1/2015 |
| case8 | Guangdong | Dongguan | 52 | F | 14/1/2015 |
| case9 | Guangdong | Donggaun | 37 | M | 14/1/2015 |
| case10 | Guangdong | Shenzhen | 66 | M | 18/1/2015 |
| case11 | Guangdong | Shanwei | 1 | F | 18/1/2015 |
| case12 | Guangdong | Heyuan | 3 | F | 19/1/2015 |
| case13 | Guangdong | Shenzhen | 58 | M | 22/1/2015 |
| case14 | Guangdong | Shantou | 68 | M | 23/1/2015 |
| case15 | Guangdong | Zhaoqing | 4 | M | 23/1/2015 |
| case16 | Guangdong | Meizhou | 46 | M | 24/1/2015 |
| case17 | Guangdong | Chaozhou | 77 | M | 24/1/2015 |
| case18 | Guangdong | Dongguan | 62 | M | 25/1/2015 |
| case19 | Guangdong | Shanwei | 78 | F | 25/1/2015 |
| case20 | Guangdong | Jieyang | 52 | M | 25/1/2015 |
| case21 | Guangdong | Shanwei | 20 | F | 26/1/2015 |
| case22 | Guangdong | Shenzhen | 75 | F | 28/1/2015 |
| case23 | Guangdong | Meizhou | 62 | F | 28/1/2015 |
| case24 | Guangdong | Meizhou | 83 | M | 28/1/2015 |
| case25 | Guangdong | Heyuan | 4 | M | 28/1/2015 |
| case26 | Guangdong | Shenzhen | 21 | M | 29/1/2015 |
| case27 | Guangdong | Foshan | 42 | F | 29/1/2015 |
| case28 | Guangdong | Meizhou | 59 | M | 29/1/2015 |
| case29 | Guangdong | Shanwei | 9 | F | 31/1/2015 |
| case30 | Guangdong | Jieyang | 48 | M | 31/1/2015 |
| case31 | Guangdong | Guangzhou | 56 | M | 3/2/2015 |
| case32 | Guangdong | Zhongshan | 0.75 | F | 3/2/2015 |
| case33 | Guangdong | Shenzhen | 77 | M | 4/2/2015 |
| case34 | Guangdong | Shenzhen | 51 | M | 5/2/2015 |
| case35 | Guangdong | Guangzhou | 48 | M | 6/2/2015 |
| case36 | Guangdong | Zhuhai | 76 | M | 6/2/2015 |
| case37 | Guangdong | Huizhou | 62 | F | 6/2/2015 |
| case38 | Guangdong | Chaozhou | 55 | M | 6/2/2015 |
| case39 | Guangdong | Zhongshan | 2 | F | 6/2/2015 |
| case40  case41 | Guangdong  Guangdong | Jiangmen  Shenzhen | 56  52 | M  M | 7/2/2015  8/2/2015 |
| case42 | Guangdong | Zhongshan | 73 | M | 8/2/2015 |
| case43 | Guangdong | Shantou | 33 | M | 10/2/2015 |
| case44 | Guangdong | Guangzhou | 61 | M | 12/2/2015 |
| case45 | Guangdong | Shantou | 60 | M | 12/2/2015 |
| case46 | Guangdong | Guangzhou | 51 | M | 13/2/2015 |
| case47 | Guangdong | Shenzhen | 58 | F | 13/2/2015 |
| case48 | Guangdong | Chaozhou | 82 | M | 13/2/2015 |
| case49 | Guangdong | Chaozhou | 61 | M | 13/2/2015 |
| case50 | Guangdong | Meizhou | 45 | M | 14/2/2015 |
| case51 | Guangdong | Guangzhou | 33 | F | 17/2/2015 |
| case52 | Guangdong | Dongguan | 65 | M | 18/2/2015 |
| case53 | Guangdong | Meizhou | 78 | M | 20/2/2015 |
| case54 | Guangdong | Shantou | 48 | F | 21/2/2015 |
| case55 | Guangdong | Meizhou | 51 | M | 22/2/2015 |
| case56 | Guangdong | Shantou | 51 | M | 22/2/2015 |
| case57 | Guangdong | Jiangmen | 52 | M | 22/2/2015 |
| case58 | Guangdong | Huizhou | 57 | M | 22/2/2015 |
| case59 | Guangdong | Heyuan | 55 | M | 22/2/2015 |
| case60 | Guangdong | Zhaoqing | 59 | M | 25/2/2015 |
| case61 | Guangdong | Heyuan | 3 | M | 25/2/2015 |
| case62 | Guangdong | Foshan | 18 | F | 25/2/2015 |
| case63 | Guangdong | Zhaoqing | 78 | M | 26/2/2015 |
| case64 | Guangdong | Shantou | 80 | M | 27/2/2015 |
| case65 | Guangdong | Foshan | 36 | M | 1/3/2015 |
| case66 | Guangdong | Dongguan | 45 | M | 1/3/2015 |
